# Growing straight through walls

**DOI:** 10.7554/eLife.61647

**Published:** 2020-09-01

**Authors:** Subramanian Sankaranarayanan, Sharon A Kessler

**Affiliations:** 1 Department of Botany and Plant Pathology, Purdue UniversityWest LafayetteUnited States; 2 Purdue Center for Plant Biology, Purdue UniversityWest LafayetteUnited States

**Keywords:** pollen tube guidance, stigma papilla, KATANIN, mechanical anisotropy, *A. thaliana*

## Abstract

The pollen tube in a flowering plant grows in a direction that is influenced by the mechanical properties of the stigma papillae and the organization of structures called cortical microtubules inside these cells.

**Related research article** Riglet L, Rozier F, Kodera C, Bovio S, Sechet J, Isabelle F, Gaude T. 2020. KATANIN-dependent mechanical properties of the stigmatic cell wall mediate the pollen tube path in Arabidopsis. *eLife* **9**:e57282. doi: 10.7554/eLife.57282

In a flowering plant, reproduction begins when grains of pollen stick to cells called stigma papillae that are located at the top of the pistil, which is the female part of the flower. A cell called a pollen tube then delivers the sperm cells contained in the pollen grains to the female gametes for fertilization. This is a long journey that involves the pollen tube travelling from the stigma papillae at the top of the pistil to the ovules that contain the female gametes, which are at the bottom of the pistil.

So how does the plant ensure that the pollen tube – which is a single cell that grows longer over time – finds the ovules and does not get lost en route? Several molecules and nutrients secreted by the pistil direct the growth of the pollen tube (Higashiyama and Takeuchi, 2015). However, the identity of the cues that guide the pollen tube in the first stages of its journey have remained a mystery.

Most plant cells grow by increasing their surface area while remaining attached to neighboring cells: pollen tubes are different in that they are tip-growing cells that can grow through the walls of other cells to reach their target. When the pollen tube first enters the pistil, it remains within the cell wall of the stigma papillae (Figure 1; left): could the components of this cell wall, or the mechanical properties of these cells, influence the growth of the pollen tube?

**Figure 1.**
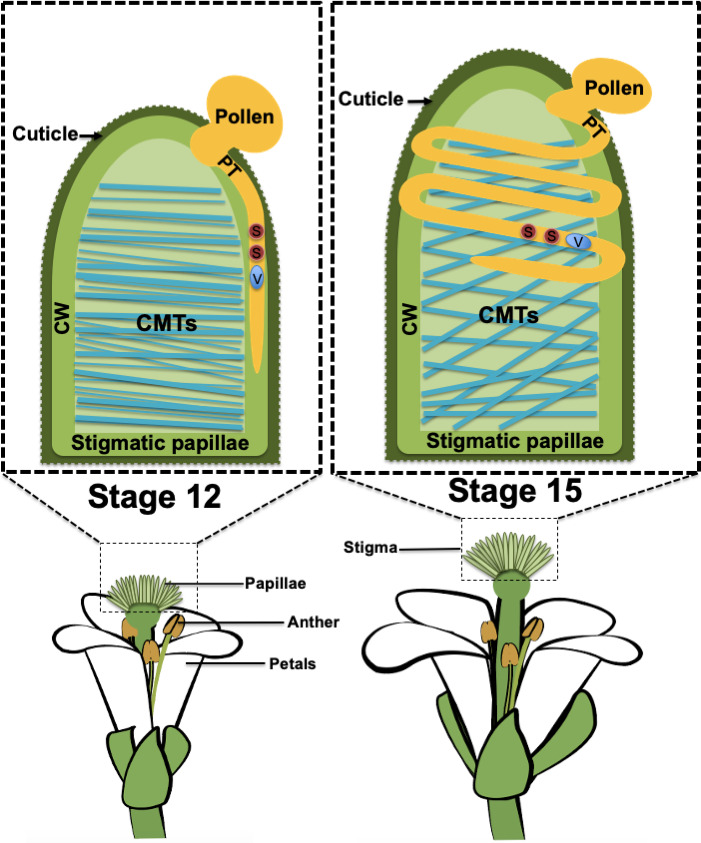
Pollen tube growth in stigma papillae. When a grain of pollen (shown in mustard) lands on a papilla in the stigma (green) of a flowering plant, a pollen tube (PT; also shown in mustard) begins to grow through the cell wall (CW) of the papilla so that the sperm cells (S; red) in the pollen can be delivered to the female gametes, which are located in ovules deep inside the plant. In stage 12 flowers (left), the organization of the cortical microtubules (CMTs; blue lines) inside the papilla is highly anisotropic and the pollen tube grows in a straight line. In older stage 15 flowers (right), the organization of the microtubules is isotropic and the pollen tube forms a coil around the papilla as it grows. The vegetative cell (**V**) makes up the body of the pollen tube and encloses the sperm cells.

A number of studies have demonstrated how mechanical properties can influence a variety of cellular processes – including proliferation, differentiation, migration and cell signaling – in animal cells (Discher et al., 2005; Fu et al., 2010; Provenzano and Keely, 2011), and there is evidence that mechanical properties can also shape plant growth and development (Eng and Sampathkumar, 2018; Sampathkumar et al., 2019). For example, it is known that when a pollen tube penetrates the cell wall of a stigma papilla, it causes changes in the mechanical properties of the cell wall by exerting pressure (Zerzour et al., 2009; Sanati Nezhad and Geitmann, 2013).

However, the role of these mechanical properties in regulating the growth of pollen tube has not been explored in detail. Moreover, although the pollen tube is a good model for understanding the behavior of plant cells, and has been used in numerous in vitro studies of tip growth, it has proved challenging to study the directed growth of pollen tubes through the cell walls of stigma papillae in vivo. Now, in eLife, Thierry Gaude and co-workers at the Université de Lyon – including Lucie Riglet as first author – report the results of experiments on the model plant *Arabidopsis thaliana* that combine the power of microscopy, genetics, and chemical biology to provide new insights into the regulation of pollen tube growth (Riglet et al., 2020).

As stigmas age, they become less receptive to pollen (Gao et al., 2018), and the observation that pollen tubes tend to coil around papillae in aging stigmas forms the basis of this study. Riglet et al. found that aging was associated with changes in the organization of the cortical microtubules in the cytoskeleton: the orientations of these microtubules were more isotopic in older stigmas than in younger stigmas (Figure 1). To test the hypothesis that the organization of these microtubules has a role in directing pollen tube growth, the researchers examined plants that had a loss of function mutation in an enzyme called KATANIN (KTN1): this enzyme can sever microtubules, and thus allows microtubules to be re-oriented following mechanical stimulation (Sampathkumar et al., 2014). Riglet et al. found that pollen tubes coiled around the papillae in both young and old mutant plants: this indicates that the arrangement of the microtubules affects the ability of pollen tubes to grow straight through the cell walls and into the rest of the pistil.

Cortical microtubules are associated with cellulose synthesis, so the researchers tested whether the stiffness and composition of the cell wall in mutant and aging papillae was associated with pollen tube coiling. They found that softer cell walls and isotropic arrangements of cellulose microfibrils in mutant and aging papillae were associated with faster pollen tube growth and loss of directionality. Overall, the latest work supports the thesis that the mechanical properties and cell wall composition of the stigma papillae have an influence on pollen tube growth and help to guide it through the stigma. Moreover, by providing fundamental insights into the process of sexual reproduction in plants, the work is also relevant in the context of global food security as pollen-stigma interactions are critical for successful pollination and seed production in flowering plants.

Apart from pollen tubes, several types of plant, animal and fungal cells grow invasively, including root hairs, fibroblasts, cancer cells and fungal hyphae. In the future, it will be important to determine the contribution of mechanical forces to invasive growth. New technological advances such as lab-on-a-chip, MEMS (micro-electro-mechanical systems), deep-tissue imaging and computational tools will help researchers to measure the mechanical forces operating on and in cells (Nezhad et al., 2013). The pollen tube/pistil system will also make it possible to explore how chemical guidance cues work together with mechanical forces to regulate directional cell growth.
